# TRIM44 promotes BRCA1 functions in HR repair to induce Cisplatin Chemoresistance in Lung Adenocarcinoma by Deubiquitinating FLNA

**DOI:** 10.7150/ijbs.71283

**Published:** 2022-04-18

**Authors:** Shuai Zhang, Mengru Cao, Shi Yan, Yuechao Liu, Weina Fan, Yimeng Cui, Fanglin Tian, Ruixue Gu, Yaowen Cui, Yuning Zhan, Yuanyuan Sun, Ying Xing, Li Cai, Yang Song

**Affiliations:** 1 The Fourth Department of Medical Oncology, Harbin Medical University Cancer Hospital, 150 Haping Road, Harbin, 150081, China.; 2 The First Department of Orthopedic Surgery, The Second Affiliated Hospital of Harbin Medical University, Harbin, 150086, China.

**Keywords:** TRIM44, lung adenocarcinoma, cisplatin chemoresistance, BRCA1, FLNA

## Abstract

Tripartite motif-containing 44 (TRIM44) has recently been implicated in various pathological processes in numerous cancers, including lung adenocarcinoma (LUAD); however, its functional roles in chemoresistance are poorly understood. Herein, TRIM44 knockdown sensitized LUAD cells to cisplatin and enhanced cisplatin-induced apoptosis. Microarray analysis indicated that the “*Role of BRCA1 in DNA damage*” and the *BRCA1* gene expression were positively regulated by TRIM44, which was further verified by immunofluorescence, qRT-PCR, and Western blotting. BRCA1 depletion effectively abolished TRIM44-modulated cisplatin resistance and regulation of homologous recombination (HR) repair. Interestingly, TRIM44 interacted with FLNA, an upstream regulator of BRCA1 as specified by *STRING* V 11.5, and facilitated its stability and deubiquitination. FLNA was also found to be required for the functions of TRIM44 in drug resistance. Using animal models, overexpression of TRIM44 was shown to confer resistance to cisplatin in a BRCA1- and FLNA-dependent manner. TRIM44 expression levels in tissues from cisplatin-resistant LUAD patients were significantly higher than those in tissues from cisplatin-sensitive LUAD patients. Collectively, our study results demonstrate that the TRIM44/FLNA/BRCA1 axis is involved in cisplatin chemoresistance, providing potential therapeutic targets for LUAD patients with cisplatin resistance.

## Introduction

Despite the fact that the incidence of lung cancer has been surpassed by that of breast cancer, lung cancer remains the leading cause of cancer-related death worldwide 1, 2. Approximately 45-55% of non-small-cell lung cancers (NSCLCs, accounting for 85% of all lung cancers) are lung adenocarcinoma (LUAD) 3. Platinum compounds, such as cisplatin [also known as cis-diamminedichloroplatinum (II), CDDP], are front-line chemotherapeutic agents for LUAD 4. Cisplatin treatment gives patients a dramatic survival advantage and works by inducing DNA-platinum adduct formation and apoptotic signaling in cancer cells; however, resistance limits its clinical utility and effectiveness in patients with LUAD 5, 6. Thus, the identification of accurate predictive markers for response or resistance and a better understanding of the molecular mechanisms of cisplatin chemoresistance are critical.

Tripartite motif (TRIM)-containing proteins are typically characterized by a RING-finger domain, one or two B-box domains, and a coiled-coil domain 7. TRIM44 is an atypical TRIM family protein that lacks an N-terminal RING-finger domain but contains a zinc finger domain found in ubiquitin hydrolases (ZF UBPs) and ubiquitin specific proteases (USPs) 8. Therefore, TRIM44 could function as a ''USP-like-TRIM” to deubiquitinate and stabilize associated proteins 8-12. TRIM44 is involved in the virus-mediated immune response, neurodegenerative diseases, developmental disorders and malignant diseases, including lung cancer 12-15. Increasing evidence indicates that TRIM44 plays pivotal roles in tumor progression, as it can potentiate the proliferation, migration and invasion of cancer cells and can induce drug resistance and radioresistance 9-11, 15-27. Regarding chemotherapy resistance, only one publication showed that TRIM44 conferred the resistance of hepatocellular carcinoma cells to doxorubicin by regulating the NF-κB signaling pathway 27. Our previous report revealed that TRIM44 increased the metastatic and proliferative capacity of lung cancer cells by inducing epithelial‑to‑mesenchymal transition and accelerating the G1/S phase transition 15; however, the functions and mechanisms of TRIM44 in LUAD chemoresistance, including cisplatin resistance, are still unclear.

Breast cancer susceptibility gene 1 (*BRCA1*), a tumor suppressor strongly associated with familial cancers, was initially cloned in 1994 28. The BRCA1 protein functions in numerous cellular and biochemical processes involved in the maintenance of chromosomal stability and tumor repression through its involvement in DNA damage-induced repair, the cell cycle, transcription, chromatin remodeling, epigenetic control, transcriptional regulation and apoptosis 29-31. In the DNA damage response (DDR), BRCA1 plays a critical role in DNA damage repair processes, including the activation of double-strand breaks (DSBs) repair. Platinum-induced DNA cross-linking can result in DNA DSBs, a leading lethal type of DNA damage 32, 33. Next, homologous recombination (HR) repair, a major system required for DNA DSBs, is induced by BRCA1 32, 33. BRCA1 promotes the recruitment of the recombinational repair protein RAD51 to damage sites 34. By searching the homologous chromatid, the generation of RAD51-coated filaments at DNA damage sites can induce DNA strand repair 33. The properties of RAD51 foci indicate the multimeric nucleoprotein complexes engaged in HR 35. It has been reported that following exposure to the DNA cross-linking agent cisplatin, BRCA1 contributes to increased chemoresistance of cancer cells 36-38. Moreover, a high BRCA1 expression level predicts the poor efficacy of cisplatin-based neoadjuvant chemotherapy in cancer patients 39. To date, the mechanism and factors that regulate the effects of BRCA1 on HR repair have not been fully elucidated.

Herein, we revealed for the first time that TRIM44 is implicated in cisplatin resistance via cell-based assays, animal models and analyses of tissue specimens derived from LUAD patients. Notably, TRIM44 knockdown enhanced the sensitivity of LUAD cells to cisplatin *in vitro* and *in vivo*. Mechanistically, TRIM44 was shown to control the effect of BRCA1 on HR repair and BRCA1 expression by increasing FLNA stability. Our findings indicate an important role of the TRIM44/FLNA/BRCA1 axis in chemoresistance in LUAD.

## Results

### TRIM44 confers cisplatin resistance in LUAD cells

**Figure 1:**
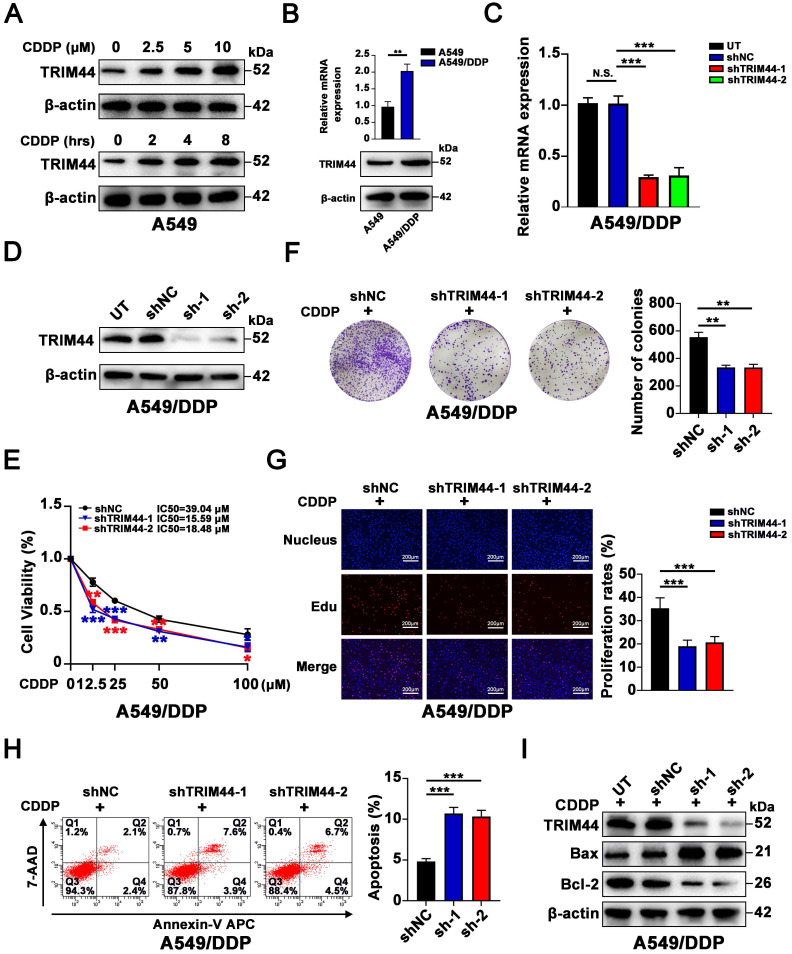
**TRIM44 knockdown reverses cisplatin resistance in cisplatin-resistant LUAD cells. (A)** The protein expression of TRIM44 after treatment with different concentrations of cisplatin for 24 h (upper panel) or 10 µM cisplatin for different time periods (lower panel) in A549 cells. **(B)** mRNA (upper panel) and protein (bottom panel) expression of TRIM44 in A549 and A549/DDP cell lines. **(C-D)** TRIM44 mRNA (C) and protein (D) expression in A549/DDP cells transfected with shNC, shTRIM44-1 and shTRIM44-2. **(E)** CCK-8 analysis showed the viability of the above cells following 48 h cisplatin treatment. **(F)** The indicated cells were treated with cisplatin for 14 days at a dosage of 10 µM. Colonies were stained with crystal violet (left panel). The bar graphs show the statistical analysis of the number of colonies (right panel). **(G)** EdU assay of shNC, shTRIM44-1 and shTRIM44-2 cells in the presence of cisplatin (10 µM). **(H)** Representative images (left panel) and bar graphs showing the statistical analysis (right panel) of Annexin V-APC/7-AAD staining of the designated cells that were treated for 24 h with 10 µM cisplatin.** (I)** Protein expression of apoptosis-related molecules. Data are shown as the mean ± SD. *P* > 0.05 was considered not significant (N.S.), *P < 0.05, **P < 0.01, and ***P < 0.001.

To investigate whether there is a link between TRIM44 expression and cisplatin resistance, cisplatin‐sensitive cells (A549 cells) were exposed to cisplatin at different concentrations for 24 h and to 10 μM cisplatin for different amounts of time. Intriguingly, cisplatin treatment significantly increased the TRIM44 expression in A549 cells in a dose- and time-dependent manner (Figure 1A). Furthermore, compared to that in cisplatin-sensitive cells (A549 cells), TRIM44 expression in cisplatin-resistant cells (A549/DDP cells) was considerably elevated (Figure 1B). Thus, A549/DDP cells were utilized for the loss-of-function model, while A549 cells were used for the gain-of-function model. We silenced TRIM44 expression in A549/DDP cells using two independent TRIM44 shRNAs. Then, we generated stable TRIM44 shRNA-expressing clones [shTRIM44-1 (also designated shTRIM44) and shTRIM44-2] and a control shRNA-negative control-expressing clone (shNC). Successful knockdown of TRIM44 was validated by qRT-PCR and Western blotting (Figure 1C-D). When treated with cisplatin at various concentrations, the viability of TRIM44 knockdown cells was lower than that of the corresponding shNC cells, as assessed by the CCK-8 assay (Figure 1E). Plate colony formation assay and EdU assay revealed that TRIM44 depletion impaired cisplatin-resistant LUAD cell proliferation (Figure 1F-G). Then, we evaluated the role of TRIM44 in cisplatin-induced apoptosis by flow cytometric analysis and Western blotting. In line with our expectations, TRIM44 knockdown dramatically enhanced the apoptosis of A549/DDP cells after treatment with cisplatin. Increased expression of the proapoptotic protein (Bax), and decreased expression of the antiapoptotic protein (Bcl-2) occurred concomitantly (Figure 1H-I).

For the gain-of-function model, qRT-PCR and Western blotting showed that A549 cells were stably transfected with TRIM44 overexpression or control vector plasmid (Figure S1A-B). Next, TRIM44-overexpressing clones (TRIM44) and a control-expressing clone (Ctrl) were established. In contrast, TRIM44 overexpression increased the chemoresistance of cisplatin-sensitive cells, as determined by the CCK-8 (Figure S1C), plate colony formation (Figure S1D) and EdU (Figure S1E) assays. As expected, high expression of TRIM44 was related to decreased apoptosis, as further proven by flow cytometric analysis (Figure S1F) and apoptotic marker evaluation (Figure S1G). Our results indicated that TRIM44 is critical for the resistance of LUAD cells to cisplatin.

### TRIM44 promotes BRCA1 expression and the effect of BRCA1 on HR repair

**Figure 2:**
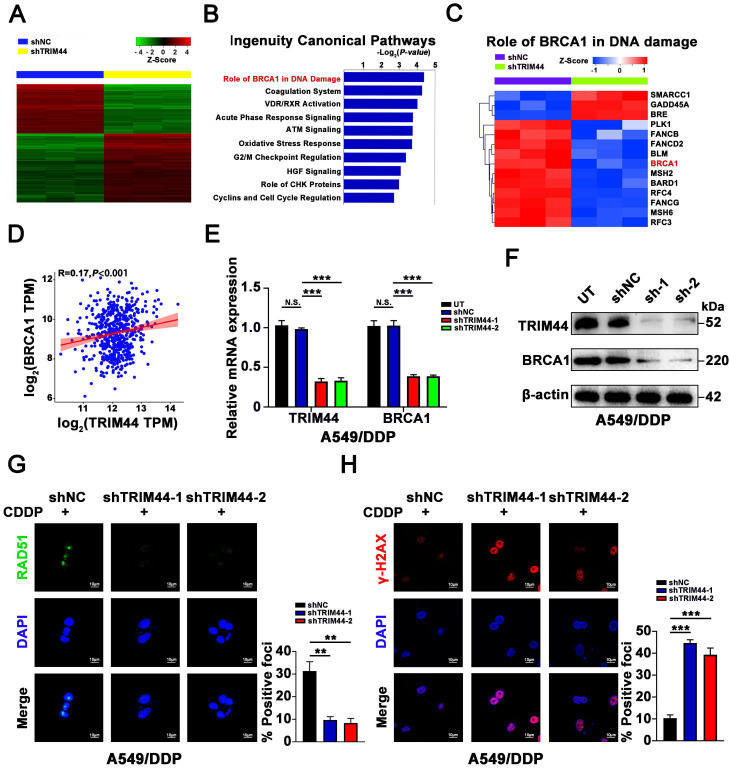
** TRIM44 knockdown inhibits BRCA1 expression and the effect of BRCA1 on HR repair. (A)** Heatmap of differentially expressed genes (DEGs) obtained from global gene expression profiling of shNC (blue) or shTRIM44 (yellow) derived from A549/DDP cells using a microarray assay. The normalized expression of genes was indicated by the Z score. **(B)** The “*Canonical Pathway Analysis*” in the IPA software was used to summarize the enrichment of DEGs in the classical signaling pathways, and all signaling pathways were ranked using -Log_2_(*P value*). **(C)** Heatmap showing DEGs of shNC (purple) or shTRIM44 (green) derived from A549/DDP cells involved in the “*Role of BRCA1 in DNA damage*”. 11 DEGs were down-regulated, including *BRCA1* (red), whereas 3 DEGs were up-regulated. **(D)** The correlation between the expression of TRIM44 and BRCA1 in LUAD samples in the TCGA database is shown. **(E-F)** The expression of BRCA1 was reduced following TRIM44 knockdown, according to qRT-PCR (E) and Western blotting (F) analysis. **(G-H)** shNC, shTRIM44-1 and shTRIM44-2 derived by A549/DDP cells were treated with 10 μM cisplatin for 24 h. (G) Representative immunofluorescence images showing RAD51 foci (left panel) and bar graphs showing the statistical analysis (right panel). (H) Representative immunofluorescence images showing γ-H2AX foci (left panel) and bar graphs showing the statistical analysis (right panel). Data are shown as the mean ± SD. *P* > 0.05 was considered not significant (N.S.), **P < 0.01, and ***P < 0.001.

To investigate the mechanism by which TRIM44 induces cisplatin resistance, microarray analysis was conducted to screen the global gene expression profiles of shNC and shTRIM44. In total, 490 upregulated and 722 downregulated genes were found after TRIM44 knockdown (Figure 2A). “*Disease or Functions Annotation*” analysis of the differentially expressed genes (DEGs) (*P* < 0.05 and absolute fold change > 2) via IPA software showed that TRIM44 might play roles in cell growth and proliferation, DNA replication, recombination, repair, and the cell cycle (Figure S2A). Moreover, “*Canonical Pathway analysis*” of IPA software revealed that multiple pathways might be regulated by TRIM44. Among them, the “*Role of BRCA1 in DNA damage*” ranked first according to the *P* value (Figure 2B). In detail, 11 genes involved in “*Role of BRCA1 in DNA damage*”, including *BRCA1*, were downregulated, whereas 3 genes were upregulated (Figure 2C). Consistently, based on The Cancer Genome Atlas (TCGA) database, Gene Ontology (GO) enrichment analysis indicated that TRIM44 functions in “*DNA repair*” (Figure S2B). TRIM44 mRNA expression was positively linked with BRCA1 mRNA expression in LUAD samples from the TCGA database (Figure 2D). In agreement with the microarray results, when TRIM44 was knocked down, the mRNA and protein levels of BRCA1 were decreased (Figure 2E-F).

Considering the role of BRCA1 in HR repair, we speculated that TRIM44 might lead to increased HR repair and decreased DNA damage. IF staining was applied to measure the formation of RAD51 and γ-H2AX foci in cisplatin-resistant cells after cisplatin treatment for 24 h. As expected, TRIM44 knockdown decreased the number of RAD51 foci (Figure 2G) but increased the number of γ-H2AX foci, which indicated DNA damage (Figure 2H). In contrast, overexpression of TRIM44 enhanced the expression of BRCA1 (Figure S2C-D). IF assays showed that, in response to cisplatin, TRIM44 overexpression induced RAD51 foci formation but reduced γ-H2AX foci development in cisplatin‐sensitive cells (Figure S2E-F). These results demonstrate that TRIM44 critically regulates the role of BRCA1 in HR repair by regulating BRCA1 expression.

### BRCA1 is required for TRIM44-induced cisplatin resistance

In concordance with previous reports that BRCA1 induced cisplatin resistance 40, 41, we also found that BRCA1 knockdown sensitizes cisplatin-resistant LUAD cells to drug and attenuates cisplatin-induced apoptosis (Figure S3).

**Figure 3:**
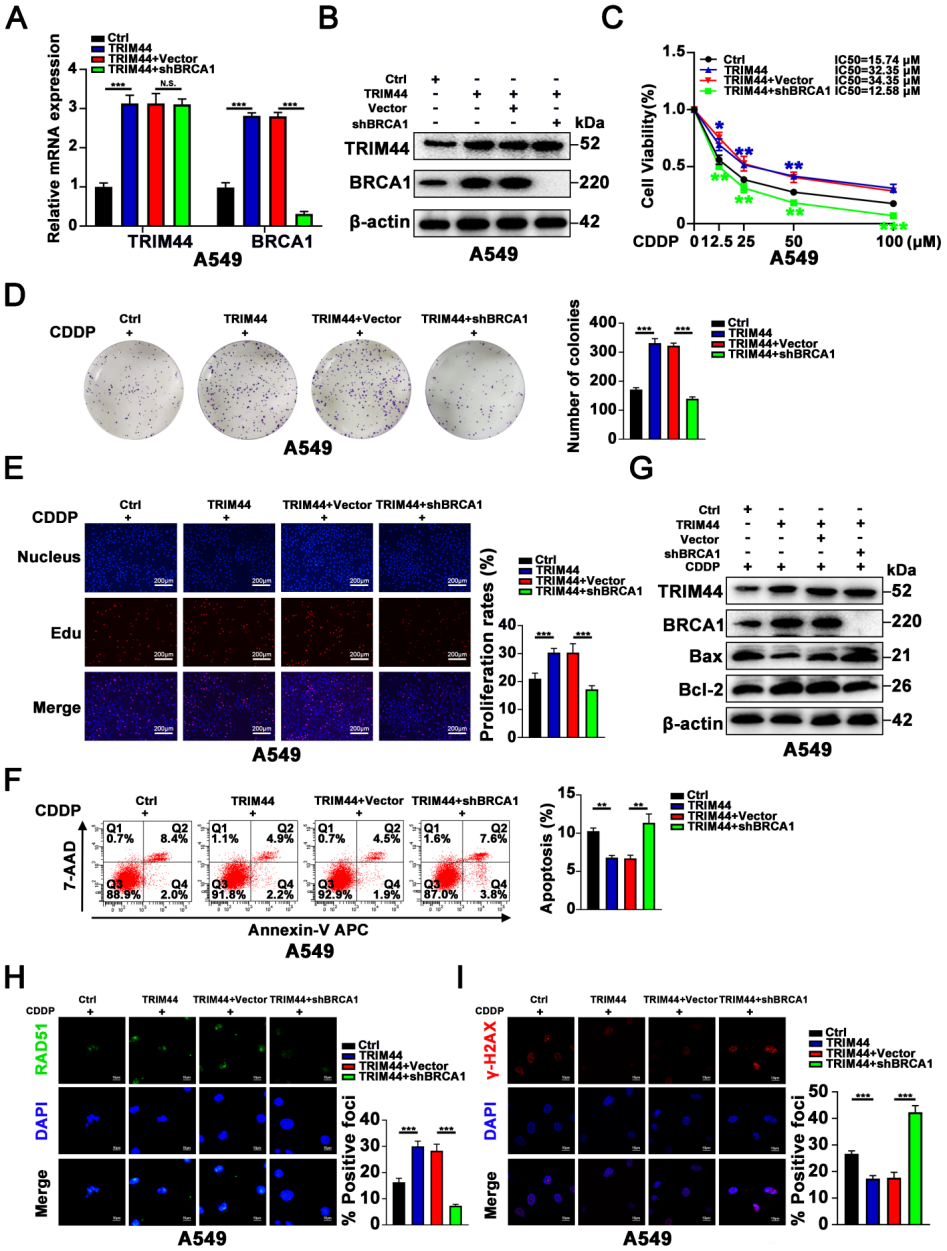
** BRCA1 is required for TRIM44-induced cisplatin resistance.** A549 cells were transfected with lentivirus expressing either Ctrl, TRIM44, TRIM44+Vector, or TRIM44+shBRCA1. **(A-B)** The expression of BRCA1 was reduced following shBRCA1 transfection into TRIM44-overexpressing A549 cells, according to qRT-PCR (A) and Western blotting (B) analysis. **(C)** CCK-8 analysis showed the viability of the indicated A549-derived cells treated with different concentrations of cisplatin. **(D-E)** The colony formation and proliferation ability of the indicated A549-derived cells after treatment with cisplatin were measured by colony formation (D) and EdU (E) assays. **(F-G)** Apoptosis analysis of the indicated A549-derived cells treated with cisplatin was performed by flow cytometric analysis (F) and Western blotting (G). **(H-I)** The indicated A549-derived cells were treated with cisplatin for 24 h. (H) Representative immunofluorescence images showing RAD51 foci (left panel) and bar graphs showing the statistical analysis (right panel). (I) Representative immunofluorescence images showing γ-H2AX foci (left panel) and bar graphs showing the statistical analysis (right panel). Data are shown as the mean ± SD. *P* > 0.05 was considered not significant (N.S.), *P < 0.05, **P < 0.01, and ***P < 0.001.

Next, we further explored whether BRCA1 is essential for TRIM44-mediated cisplatin resistance. shBRCA1 was transfected into TRIM44-overexpressing A549 cells, and the knockdown efficacy was confirmed by qRT-PCR and Western blotting (Figure 3A-B). We found that silencing BRCA1 dramatically reduced the promotional effects of TRIM44 on A549 cell viability, colony forming ability, and proliferation in response to cisplatin as determined by the CCK-8 (Figure 3C), colony formation (Figure 3D) and EdU (Figure 3E) assays. Apoptosis analysis by flow cytometry and Western blotting indicated that BRCA1 depletion almost abolished TRIM44-modulated apoptosis in response to cisplatin (Figure 3F-G). Consistent with our above results, IF assays demonstrated that BRCA1 knockdown impaired the TRIM44-mediated increases in HR repair marker foci (RAD51 foci per cell) formation and decreases in DNA damage marker foci (γ-H2AX foci per cell, Figure 3H-I). These data indicated that TRIM44 regulates LUAD chemoresistance in a BRCA1-dependent manner.

### TRIM44 physically binds to FLNA and regulates its stability

**Figure 4:**
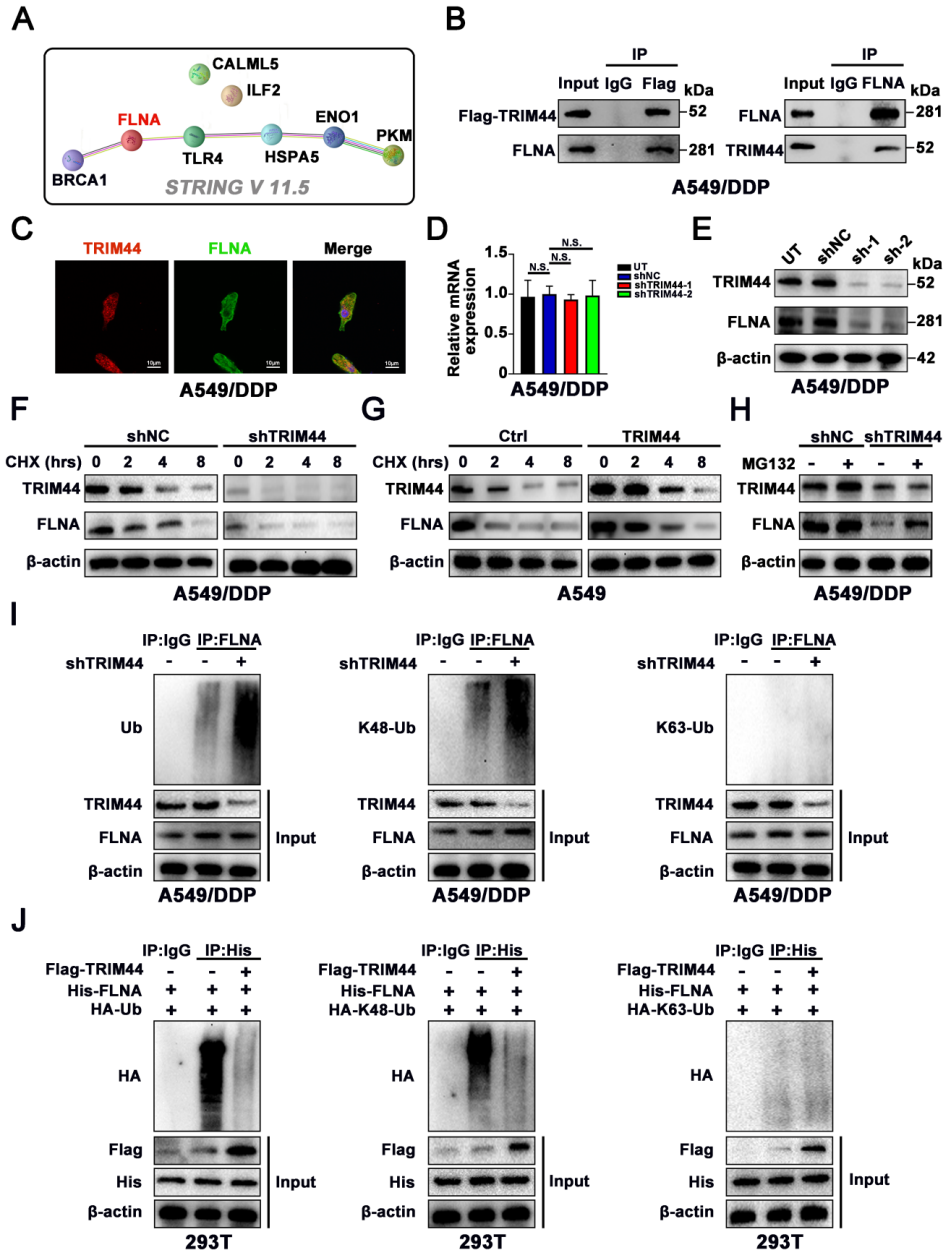
** TRIM44 physically binds to FLNA and regulates its stability. (A)** The relationship of BRCA1 and 7 potential binding proteins was assessed by *STRING* V *11.5.* **(B)** Flag-TRIM44 was transfected into A549/DDP cells, and then an IP assay revealed the association between TRIM44 and FLNA. **(C)** Immunofluorescence analysis showed the colocalization of TRIM44 and FLNA protein in A549/DDP cells. **(D-E)** qRT-PCR (D) and Western blotting (E) analysis showed the expression of FLNA after TRIM44 knockdown in A549/DDP cells. **(F)** shNC and shTRIM44 derived from A549/DDP cells were treated with cycloheximide (CHX) for different time periods. Western blotting analysis showed FLNA protein levels at different time points. **(G)** Within a specified time, Ctrl and TRIM44 derived from A549 cells were incubated with CHX. The protein levels of FLNA at different time points were detected by Western blotting. **(H)** shNC and shTRIM44 derived from A549/DDP cells were treated with or without MG132 (10 μM) for 6 h. Then, Western blotting showed FLNA protein levels. **(I)** Western blotting analysis showed the total ubiquitination, K48-linked ubiquitination, or K63-linked ubiquitination of FLNA in shNC and shTRIM44 derived from A549/DDP cells. **(J)** The indicated plasmids were cotransfected into 293T cells, and the ubiquitin status of FLNA was determined using an immunoprecipitation test. Data are shown as the mean ± SD. *P* > 0.05 was considered not significant (N.S.).

To elucidate the mechanism by which TRIM44 orchestrates BRCA1-mediated HR repair and cisplatin resistance, we reviewed all publications linked to TRIM44 in cancer. Only one publication reported proteomic analysis of TRIM44 or its partner. Wei *et al.* 10 analyzed the binding partners of TRIM44 by using a combination of Co-IP and mass spectrometry in a study on melanoma progression, and seven overlapping proteins (TLR4, ILF2, ENO1, CALML5, PKM, HSPA5, and FLNA) were identified in the two cell lines. Next, *STRING* V 11.5. (https://cn.string-db.org/) was employed to estimate the relationship between BRCA1 and these 7 candidate proteins. Of these seven proteins, only actin-binding protein filamin A (FLNA) showed a potential association with BRCA1 (Figure 4A). According to documented reports, FLNA is able to interact with BRCA1 to regulate its expression 42, 43.

Then, to further elucidate how TRIM44 interacts with FLNA, we immunoprecipitated Flag-TRIM44, and Western blotting against FLNA and TRIM44 confirmed that FLNA interacted with TRIM44, while TRIM44 was discovered after the IP of FLNA, suggesting that TRIM44 interacted with FLNA (Figure 4B). Moreover, confocal laser scanning microscopy showed colocalization of TRIM44 and FLNA in cisplatin-resistant cells (Figure 4C). To study the regulatory mechanism, we examined whether TRIM44 silencing could affect FLNA expression. As demonstrated in Figure 4D-E, the FLNA protein level was reduced following TRIM44 knockdown, while the mRNA level remained unchanged, demonstrating that TRIM44 regulates FLNA at the posttranscriptional level.

TRIM44 was reported to act as a deubiquitinating enzyme to regulate protein expression 9, 11. Cycloheximide (CHX) assays revealed that knockdown of TRIM44 decreased the stability of the FLNA protein (Figure 4F), whereas compared to Ctrl cells, TRIM44 cells showed a longer FLNA half-life (Figure 4G). As expected, we found that MG132, a proteasome inhibitor, restored FLNA protein expression, which was repressed by TRIM44 knockdown (Figure 4H). Furthermore, silencing TRIM44 enhanced the total and K48-linked ubiquitination of endogenous FLNA but had no impact on K63-linked ubiquitination in A549/DDP cells (Figure 4I). TRIM44 overexpression inhibited the overall polyubiquitination and the K48-linked polyubiquitin chain of FLNA but did not change the K63-linked polyubiquitin chain of FLNA according to ubiquitination-based IP assay results (Figure 4J). These findings suggest that TRIM44 physically interacts with FLNA, preventing FLNA degradation by regulating its deubiquitination.

### FLNA knockdown sensitizes cisplatin-resistant LUAD cells to cisplatin

The roles of FLNA in cisplatin remain largely unclear 44, 45. In the present study, we employed two distinct shRNAs targeting FLNA and a nontarget shRNA as a control to knock down FLNA in A549/DDP cells to further determine the biological function of FLNA in cisplatin chemoresistance. The knockdown efficiency of FLNA was investigated using qRT-PCR and Western blotting (Figure S4A-B). With cisplatin treatment, FLNA depletion reduced the viability, colony forming ability, and proliferation of cisplatin-treated A549/DDP cells, as suggested by the CCK-8, colony formation and EdU assays (Figure S4C-E). The role of FLNA in cisplatin-induced apoptosis was then investigated using flow cytometry and Western blotting. Knockdown of FLNA increased the cisplatin-induced apoptosis of A549/DDP cells (Figure S4F-G). Our data indicated that FLNA is a potential key driver of chemoresistance in LUAD.

### FLNA is required for TRIM44-induced cisplatin chemoresistance

**Figure 5:**
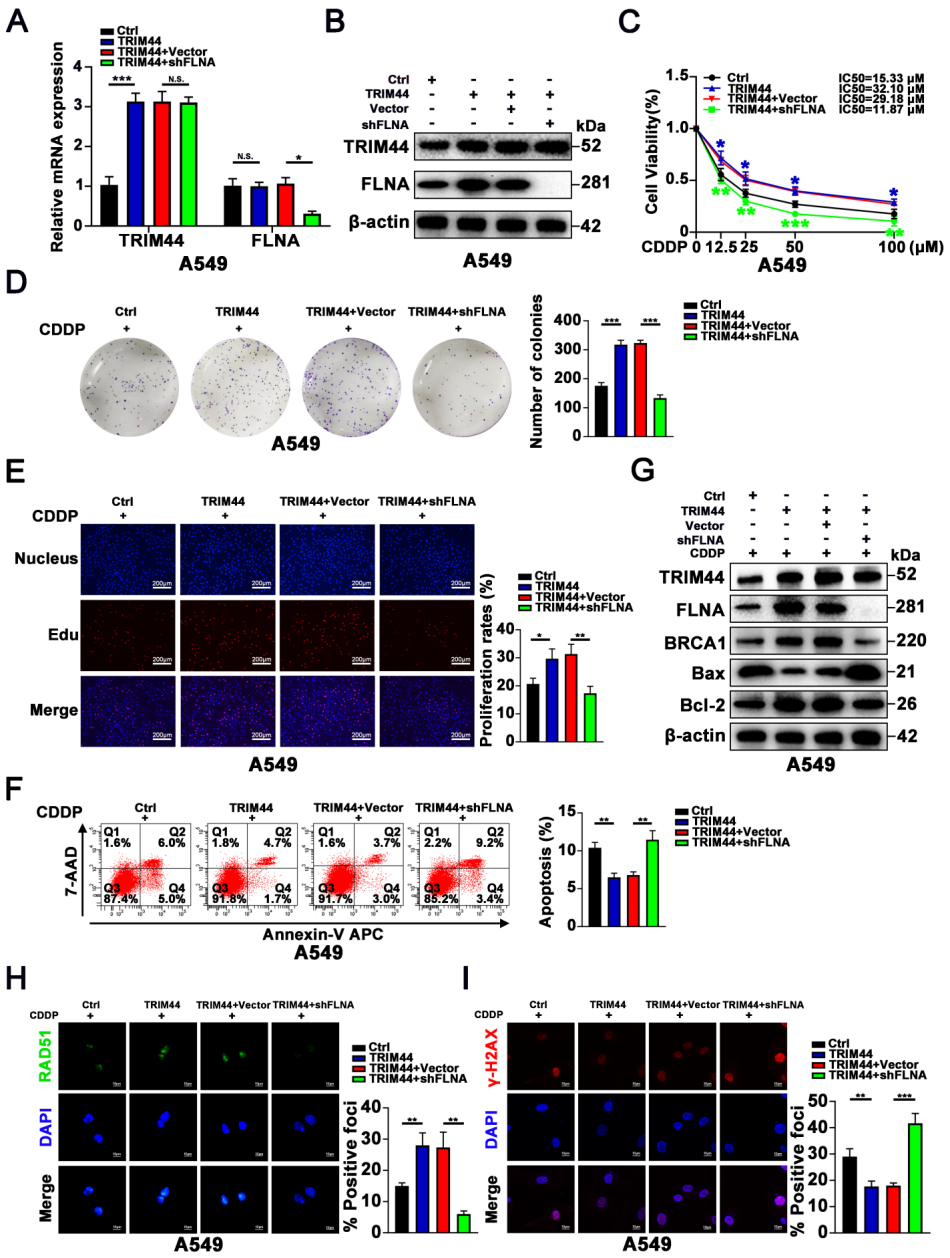
** FLNA is essential for TRIM44-induced cisplatin resistance.** A549 cells were transfected with lentivirus expressing either Ctrl, TRIM44, TRIM44+Vector, or TRIM44+shFLNA constructs. **(A-B)** qRT-PCR (A) and Western blotting (B) analyses were performed to evaluate FLNA expression levels when shFLNA was transfected into TRIM44-overexpressing A549 cells. **(C)** CCK-8 analysis showed the effect of FLNA knockdown on the viability of TRIM44-overexpressing A549 cells. **(D-E)** Colony formation analysis (D) and EdU assay (E) were used to assess the colony formation and proliferation ability of the indicated A549-derived cells. **(F-G)** Flow cytometric analysis (F) and Western blotting (G) were utilized to examine the apoptosis of the indicated A549-derived cells with cisplatin treatment. **(H-I)** The indicated A549-derived cells were treated with cisplatin for 24 h. (H) Representative immunofluorescence images showing RAD51 foci (left panel) and bar graphs showing the statistical analysis (right panel). (I) Representative immunofluorescence images showing γ-H2AX foci (left panel) and bar graphs showing the statistical analysis (right panel). Data are shown as the mean ± SD. *P* > 0.05 was considered not significant (N.S.), *P < 0.05, **P < 0.01, and ***P < 0.001.

Next, we further investigated if FLNA plays a role in TRIM44-induced cisplatin resistance. We knocked down FLNA in TRIM44-overexpressing A549 cells (TRIM44), and the knockdown efficiency was verified by qRT-PCR and Western blotting (Figure 5A-B). The CCK-8 assay demonstrated that the depletion of FLNA inhibited the effect of TRIM44 on cell viability under cisplatin stimulation (Figure 5C). The enhanced colony forming ability and cell proliferation of A549 cells generated by TRIM44 overexpression were decreased by FLNA knockdown, as demonstrated by colony formation and EdU assays (Figure 5D-E). Apoptosis assays demonstrated that FLNA depletion reversed the effect of TRIM44-attenuated cisplatin-induced cell apoptosis (Figure 5F-G). Furthermore, suppression of FLNA almost completely abolished the TRIM44-regulated role of BRCA1 in DNA damage repair, which was demonstrated by the examination of RAD51 and γ-H2AX foci (Figure 5H-I). These findings support the hypothesis that the TRIM44-induced chemoresistance of LUAD cells to cisplatin depends on FLNA. In TRIM44-depleted LUAD cells, we found that FLNA overexpression promoted BRCA1 expression (Figure S5A-B).

### TRIM44 knockdown enhances the sensitivity of xenograft tumors to cisplatin treatment

**Figure 6:**
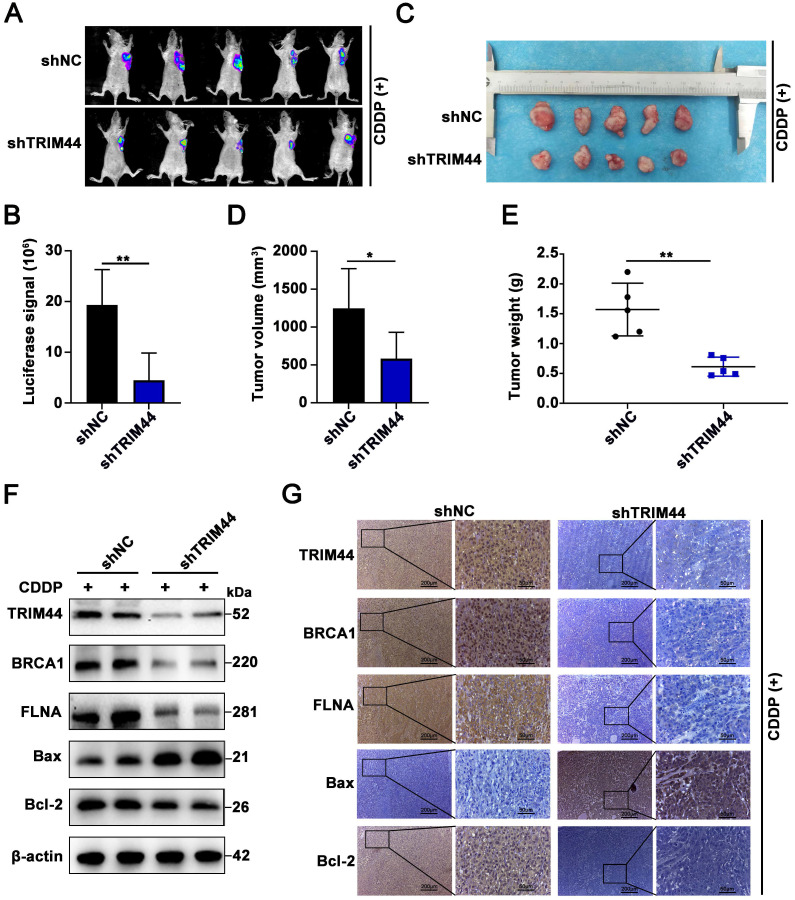
** TRIM44 knockdown enhances the sensitivity of xenograft tumors to cisplatin treatment. (A)** Representative bioluminescence images of xenograft tumors generated from shNC or shTRIM44 cells at 21 days after drug injection. **(B)** Bar graphs showing the statistical analysis of the luciferase signal in xenograft tumors. **(C)** Images of xenograft tumors in the designated group. **(D-E)** The average tumor volume (D) and weight (E) of different groups were statistically analyzed. **(F)** Western blotting was used to evaluate the protein expression levels of TRIM44, BRCA1, FLNA, Bax and Bcl-2 in harvested tumor tissues.** (G)** IHC analysis of the indicated proteins in xenograft tumor tissues. Data are shown as the mean ± SD. *P < 0.05, **P < 0.01.

To confirm the TRIM44 silencing-induced sensitive phenotype *in vivo*, we established A549/DDP xenografts using nude athymic mice. Nude mice were injected subcutaneously with shTRIM44 and NC cells. After the tumors grew to 100 mm^3^, the mice were treated with cisplatin at a dose of 5 mg/kg and injected intraperitoneally every 3 days. At 21 days after the drug injection, luciferase-expressing xenograft tumors generated from shTRIM44 cells exhibited lower bioluminescence after injection with D-luciferin than those from NC cells (Figure 6A-B). The mice were then killed, and all xenograft tumors were surgically removed, measured, and weighed. Compared with the NC group, mice injected with shTRIM44 cells had substantially decreased tumor volumes and weights (Figure 6C-E). In addition, the body weights of mice in the NC and shTRIM44 groups were not changed significantly (Figure S6A). Western blotting and IHC analyses of xenograft tumor tissues showed that Bax, a proapoptotic protein, was expressed at much higher levels in the shTRIM44 group than in the NC group, whereas the expression levels of the Bcl-2 were decreased, suggesting that TRIM44 knockdown promoted cisplatin-induced apoptosis and reversed chemoresistance (Figure 6F-G). The spontaneous xenograft tumors formed by shTRIM44 cells exhibited significantly lower levels of BRCA1 and FLNA than those formed by NC cells (Figure 6F-G). Our results indicated that TRIM44 depletion might enhance the sensitivity of xenograft tumors to cisplatin treatment by regulating the FLNA/BRCA1 axis *in vivo*.

### TRIM44 promotes cisplatin resistance via the FLNA/BRCA1 axis *in vivo*

**Figure 7:**
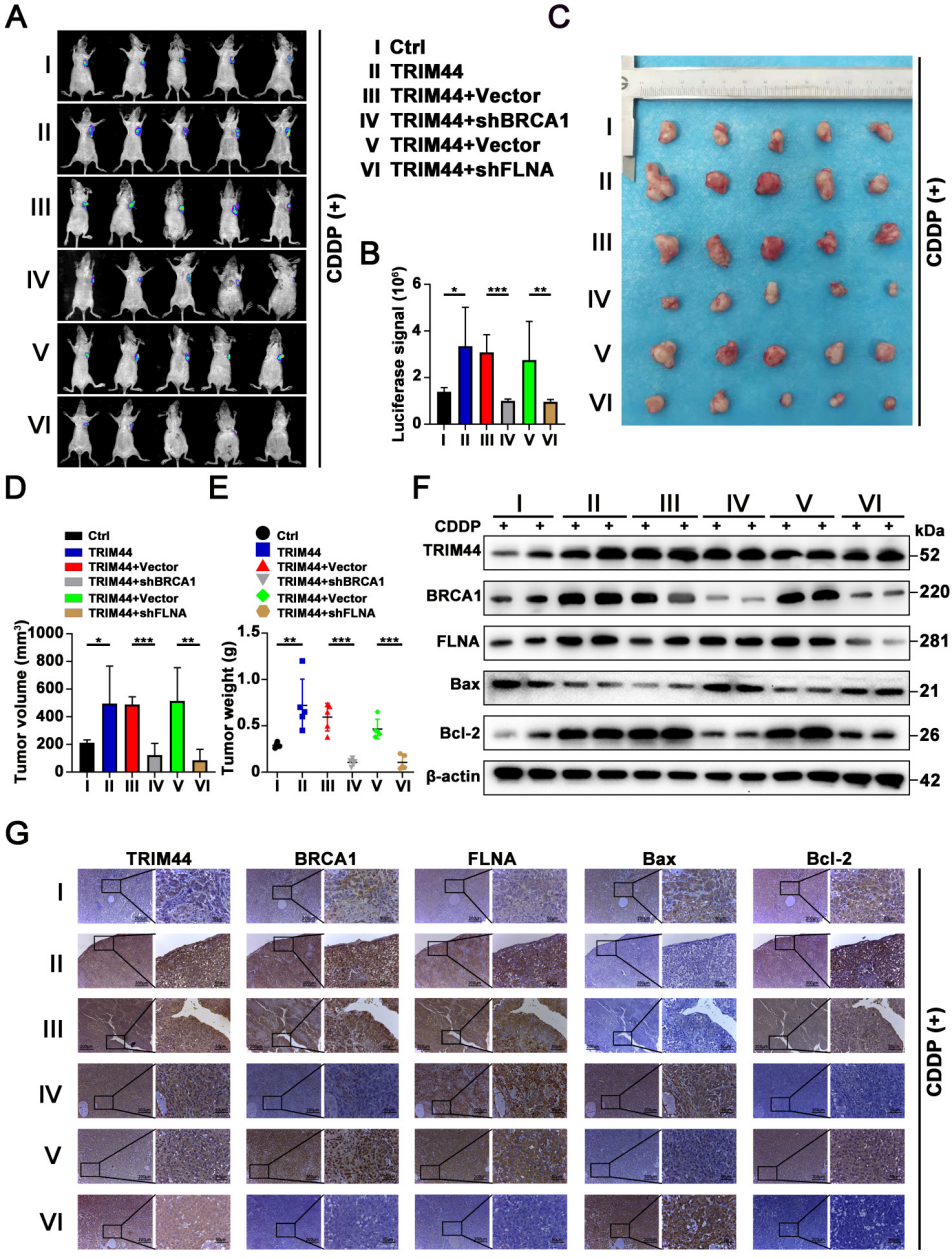
** TRIM44 promotes cisplatin resistance via the FLNA/BRCA1 axis *in vivo.*** The nude mice were randomly distributed into 6 groups: (I) Ctrl, (II) TRIM44, (III) TRIM44+Vector, (IV) TRIM44+shBRCA1, (V) TRIM44+Vector, and (VI) TRIM44+shFLNA. **(A)** Representative bioluminescence images of xenograft tumors from group I to group VI at 21 days after cisplatin injection. **(B)** Bar graphs showing the statistical analysis of the luciferase signal in xenograft tumors from group I to group VI. **(C)** Images of xenograft tumors formed in nude mice from group I to group VI at the end of the trial.** (D-E)** Statistical assessment of the mean tumor volume (D) and weight (E) in the different groups. **(F-G)** Western blotting (F) and IHC analysis (G) of the indicated proteins. Data are shown as the mean ± SD. *P < 0.05, **P < 0.01 and ***P < 0.001.

To obtain direct evidence that TRIM44 induces drug resistance to cisplatin by regulating BRCA1 and FLNA* in vivo*, nude mice were randomly distributed into 6 groups: (I) Ctrl, (II) TRIM44, (III) TRIM44+Vector, (IV) TRIM44+shBRCA1, (V) TRIM44+Vector, and (VI) TRIM44+shFLNA. Mice were treated with cisplatin and the other agents as described above. Compared with nude mice injected with Ctrl cells, nude mice injected with TRIM44 cells had an increased tumor burden (Figure 7A-E), suggesting that TRIM44 enhanced cisplatin resistance. Intriguingly, when compared to group III, group IV exhibited a significantly reduced tumor burden, indicating that BRCA1 is required for TRIM44-induced cisplatin resistance* in vivo* (Figure 7A-E). Moreover, FLNA knockdown weakened chemoresistance derived from TRIM44 cells, which was determined by the result of comparisons of experimental data from the (Ⅴ) and (Ⅵ) groups (Figure 7A-E). We also found no significant difference in the weights of the mice in the different groups (Figure S6B). Western blotting and IHC assays showed that the expression levels of FLNA, BRCA1, and Bcl-2 in tumors formed by TRIM44 cells were much higher than those formed by Ctrl cells, but the expression levels of Bax were significantly lower (Figure 7F-G). Silencing either BRCA1 or FLNA almost eliminated the effect of TRIM44 cells on cisplatin, which was demonstrated by Bcl-2 and Bax protein expression (Figure 7F-G). These findings support the conjecture that TRIM44 causes cisplatin chemoresistance by inhibiting apoptosis in a BRCA1- and FLNA-dependent manner* in vivo*.

### TRIM44 is correlated with BRCA1 and FLNA in clinical LUAD specimens

**Figure 8:**
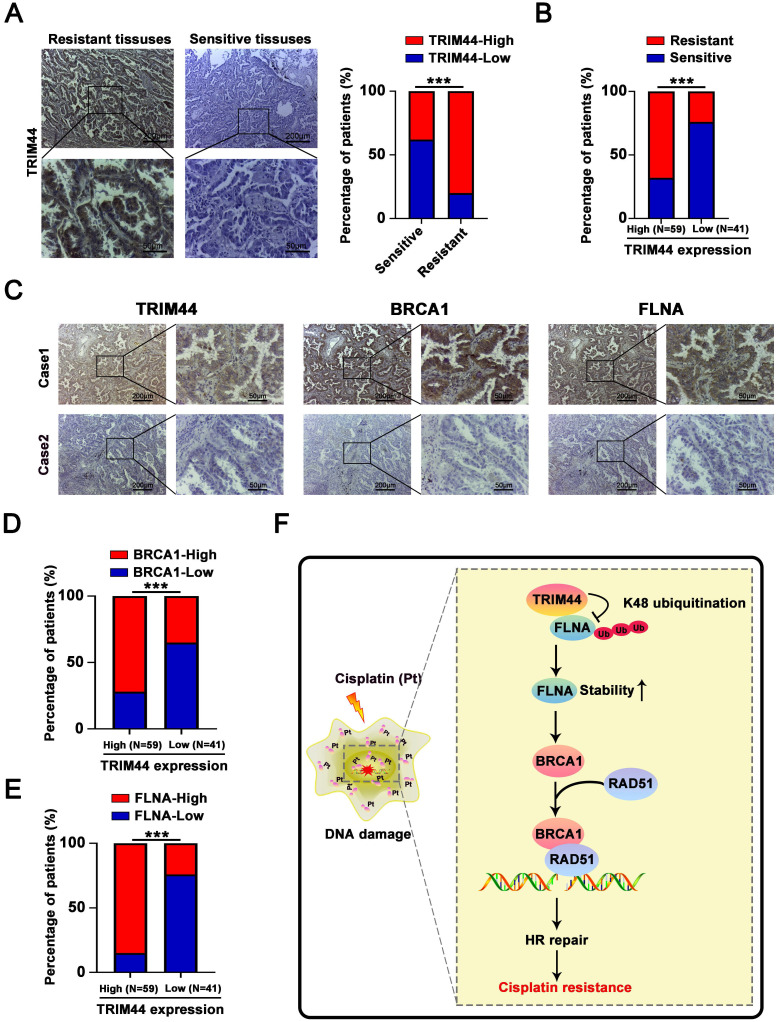
** Associations between the levels of TRIM44, FLNA, and BRCA1 in LUAD patient tissues. (A)** Characteristic IHC images of TRIM44 in cisplatin-sensitive and cisplatin-resistant LUAD tissues from the cisplatin-resistant group (PFS < 6 months) and the cisplatin-sensitive group (PFS ≥ 6 months). The percentages of patients with high expression (red bar) and low expression of TRIM44 (blue bar) were assigned according to different responses to cisplatin (right panel). **(B)** The percentages of cisplatin-resistant (red bar) and cisplatin-sensitive (blue bar) group patients according to TRIM44 low or high expression. **(C)** IHC staining for TRIM44, BRCA1, and FLNA in serial slices of LUAD tissues from two patients (representative photos). Case 1 is a LUAD patient with a high TRIM44 expression level, whereas Case 2 is a LUAD patient with low TRIM44 expression, representatively. **(D-E)** IHC analysis of LUAD tissues revealed a favorable correlation between TRIM44 expression and BRCA1 and FLNA expression. Data are shown as the mean ± SD. ***P < 0.001.

To understand whether there is a relationship between TRIM44 expression and cisplatin resistance in the clinic, LUAD tissues were collected from LUAD patients who had been treated with cisplatin. IHC arrays showed that the TRIM44 expression level in the cisplatin-resistant group (PFS < 6 months) was higher than that in the cisplatin-sensitive group (PFS ≥ 6 months, Figure 8A-B), suggesting that high TRIM44 expression in clinical LUAD specimens is significantly linked to chemoresistance. To explore the mechanisms by which TRIM44 is associated with cisplatin, IHC staining of TRIM44, BRCA1 and FLNA was performed. The distribution and intensity of TRIM44 were positively related to BRCA1 and FLNA (Figure 8C). TRIM44 expression was also correlated with the BRCA1 and FLNA expression in LUAD tissue specimens (Figure 8D-E). These data confirmed our findings in LUAD cell lines and xenograft models.

## Discussion

Drug resistance is the major cause of chemotherapy failure and disease relapse 46. Thus, the identification of determinants and understanding of mechanisms linked to LUAD cisplatin resistance are indispensable. It has been previously reported that TRIM44 can enhance the chemoresistance of hepatocellular carcinoma cells to doxorubicin by accelerating the activation of NF-κB 27. Here, we propose a working model underlying the roles of TRIM44 in modulating cisplatin resistance (Figure 8F). TRIM44 is significantly upregulated in cisplatin-resistant LUAD. In response to cisplatin, overexpressed TRIM44 interacts with FLNA and decreases the K48-linked ubiquitination of FLNA, leading to enhanced FLNA stability. FLNA upregulation promotes the expression and function of BRCA1. Then, BRCA1 recruits RAD51 and thus increases HR repair activity, eventually inducing cisplatin resistance in LUAD cells (Figure 8F).

In this study, we observed an interesting phenomenon that in which the abundance of TRIM44 increased following treatment with different concentrations of cisplatin. Cisplatin or other factors-induced DSBs is considered the most cytotoxic type of DNA damage 32, 33. The changing patterns of chromatin remodeling and posttranslational modifications, including N6-methyladenosine (m6A) modification, are pivotal for proficient DSB repair 47, 48. In response to DSBs, ATM-mediated phosphorylation at S43 activates methyltransferase 3 (METTL3), and METTL3 modulates the accumulation of DNA-RNA hybrids at DSB sites, leading to the recruitment of RAD51 and BRCA1 for HR repair 48. Overexpression of YTHN6-methyladenosine RNA binding protein 1 (YTHDF1), an N6-methyladenosine modification (m6A) reader, rescues the DSB DNA damage response 49. A previous study demonstrated that TRIM44 is transcriptionally upregulated by YTHDF1 50. In the DDR, whether the DSB-induced abundance of TRIM44 is dependent on YTHFF or other m6A enzymes requires further investigation.

Here, we revealed for the first time that TRIM44 induces cisplatin resistance in LUAD. The functional mechanisms of cisplatin chemoresistance are mainly classified into the following categories: reduced intracellular accumulation of cisplatin, increased DNA adduct tolerance, increased DNA damage repair, inhibition of apoptotic pathways, production of antioxidants and activation of autophagy 51-55. Notably, a recent study revealed that TRIM44 induced autophagy by promoting sequestosome 1/p62 oligomerization 13. This is consistent with our bioinformatic GO enrichment analysis results based on the TCGA database (Figure S2B). It would be interesting to further investigate whether TRIM44 regulates autophagy to induce cisplatin resistance in LUAD.

Our lab also first discovered that TRIM44 promotes BRCA1 expression and functions in HR repair. Accumulating evidence indicates that BRCA1 affects cellular responses to DNA damage not only by directly affecting DNA repair but also by playing a role in cell cycle checkpoint control 56. To allow DNA repair, G2/M arrest after DNA damage prevents the cell cycle from progressing to mitosis upon the induction of DNA damage 57, 58. In line with our microarray analysis (Figure 2C), BRCA1-deficient cells exhibited defective arrest at the G2/M checkpoint in response to ionizing radiation 57. It would be innovative and interesting to further determine whether TRIM44 induces BRCA1-induced G2/M phase arrest in the future.

In a very recent report, Lin *et al.* showed that TRIM44 increased nuclear FLNA expression and stability, likely through p62 11, but whether TRIM44 directly mediated FLNA stability was not elucidated. Herein, we discovered a previously unrecognized role of TRIM44 in regulating K48-linked ubiquitination targeting the FLNA protein. Ubiquitination is a common posttranslational modification, and the most well-known forms are K48-linked polyubiquitination and K63-linked polyubiquitination. Functionally, K48-linked polyubiquitination can label substrates for proteasomal degradation, whereas K63-linked polyubiquitination primarily activates signaling proteins to promote signal transduction 59, 60. A previous study reported that TRIM44 stabilizes VISA by preventing its ubiquitination and degradation, thereby promoting antiviral responses 12. In quiescent multiple myeloma cells, TRIM44 stabilizes HIF-1α, which stimulates cancer cell proliferation and survival in a hypoxic niche 9. TRIM44 can directly bind to and stabilize TLR4 to activate the AKT/mTOR pathway 10. Lin *et al.* also showed that TRIM44 can deubiquitinate p62 upon irradiation, leading to an increase in DNA damage repair 11. As a deubiquitinase, whether TRIM44 can protect the potential binding partners (*i.e.,* ILF2, ENO1, CALML5, PKM, and HSPA5) and other proteins from ubiquitin and the relative roles of TRIM44 are worth exploring.

In summary, our work uncovers a hitherto unappreciated role of TRIM44 in LUAD cisplatin chemoresistance by cell-based assays, mouse models and clinical samples. Mechanistically, TRIM44 deubiquitinates FLNA and enhances its stability to promote the expression of BRCA and its effect on HR repair, eventually inducing chemoresistance to cisplatin. Targeting the TRIM44/FLNA/BRCA1 axis may be a possible therapeutic approach to improve the outcomes of LUAD patients with resistance to cytotoxic DNA-damaging agents.

## Materials and methods

### Cell culture

A LUAD cell line resistant to cis-diamminedichloroplatinum (II) (A549/DDP) was established and applied in our previous studies 61-63. Treated with RPMI 1640 media containing 10% FBS, the human LUAD cell line A549 and A549/DDP were cultivated. In DMEM with 10% FBS, human embryonic kidney cell line (HEK-293T) was cultured. All cultures were placed in an atmosphere at 37 °C with 5% CO_2_.

### Cell transfection and stable cell lines

LUAD cells were transfected with lentiviruses expressing TRIM44 knockdown or overexpression sequences synthesized by Gene Chemistry (Shanghai, China) and Hanbio (Shanghai, China), respectively, and puromycin was used to screen them. The shRNAs of BRCA1 and FLNA were also purchased from Gene Chemistry. The detailed sequences are shown in Table S1.

Stable TRIM44 shRNA-expressing [shTRIM44-1 (shTRIM44) and shTRIM44-2] clones and a control shRNA-negative control-expressing clone (shNC) were established according to the manufacturer's protocol. A recombinant lentiviral vector expressing HBLV-h-TRIM44-3xflag-Zs-PURO was used to establish TRIM44-overexpressing clones (TRIM44). The negative control lentiviral vector HBLV-Zs-PURO was used to establish a control-expressing clone (Ctrl). shBRCA1 was stably transfected into TRIM44-overexpressing A549 cells to establish clones (TRIM44+shBRCA1). shFLNA was stably transfected into TRIM44-overexpressing A549 cells to generate clones (TRIM44+shFLNA). These two clones had the same corresponding controls (TRIM44+Vector). Puromycin was used to screen all stable cell lines.

### Quantitative real-time PCR (qRT-PCR)

Briefly, total RNA was extracted from the cells using a Total RNA Kit I (R6834-01, Omega Bio-Tek, USA). Complementary DNA was synthesized using a Transcriptor cDNA Synthesis kit (04379012001, Roche, Germany). qRT-PCR was conducted with the 7500HT Fast Real-Time PCR System (Applied Biosystems). All primer sequences are presented in Table S2.

### Western blotting

By RIPA lysis buffer (SW104-02, Sevenbio, Beijing, China) containing a proteinase inhibitor cocktail, total protein was extracted from LUAD cells and xenograft tumor tissues and measured with the BCA Protein Assay Kit (23227, Thermo Fisher Scientific, USA). Using sodium dodecyl sulfate-polyacrylamide gel electrophoresis (SDS-PAGE), the protein samples were separated. Protein on the gels was electrophoretically transferred to a PVDF membrane, which was subsequently treated with the indicated antibodies overnight at 4 °C. The next day, membranes were incubated with secondary antibody for 1 h. Finally, an enhanced chemiluminescence (ECL) detection kit (M2301, HaiGene, China) was applied to detect proteins. In Table S3, the antibodies applied in our study are presented.

### Resistance assays

To assess the viability of LUAD cells treated with cisplatin, a Cell Counting Kit-8 (CCK-8) assay was performed using a kit (CK04, Dojindo, Japan). The absorbance was detected at a wavelength of 450 nm. In addition, LUAD cells were grown at a density of 1000 cells per well in six-well plates and incubated for 14 days for the plate colony formation assay. After being fixed with formaldehyde, the colonies were stained by applying crystal violet. The count of colonies was got following washing with PBS. EdU assay was employed using the EdU Assay Kit (C10310-1, RiboBio, China) to assess DNA synthesis, indicating the proliferative capacity of the cells. Fluorescence microscopy was used to image stained LUAD cells. Resistance assays were performed with the indicated dose cisplatin treatment.

### Apoptosis analysis

To quantify apoptotic cells, LUAD cells were stained by an Annexin V-APC/7-AAD Apoptosis Detection Kit (A6030, US Everbright, China) after being treated with cisplatin for 48 h. The stained cells were quantified according to the manufacturer's instructions using flow cytometry. Anti-Bax and anti-Bcl-2 antibodies were applied to perform Western blotting for apoptosis analysis.

### Microarray analysis

Total RNA was extracted from shNC and shTRIM44 cells as described above. An Affymetrix GeneChip Human Transcriptome Array was performed as described previously 64. “*Disease or Functions Annotation*” and “*Canonical Pathway analysis*” in Ingenuity Pathway Analysis (IPA) software (version 2018; Ingenuity Systems; QIAGEN) were employed to determine the enrichment of differentially expressed genes (DEGs).

### Immunofluorescence (IF) and foci formation assays

The IF assay was conducted as previously described 65. For the RAD51 and γ-H2AX foci formation assay, the indicated cells were treated with 10 μM cisplatin to induce cellular DNA damage for 24 h, and the following steps were performed as IF assay. Anti-RAD51 and anti-γ-H2AX were applied as primary antibodies. RAD51 and γ-H2AX foci were observed using an inverted fluorescence microscope. All the antibodies used are presented in Table S3.

### Immunoprecipitation (IP) and ubiquitination assays

IP analysis was carried out as previously reported 62. The cells overexpressing flag-tagged TRIM44 were fully lysed with IP lysis buffer, and the protein was then immunoprecipitated with Flag antibody or FLNA antibody. Rabbit IgG was applied as a negative control. Western blotting was employed to examine the bound proteins.

For the ubiquitination assay, the indicated plasmids were transfected into 293T cells to directly detect the enriched total ubiquitinated, Lys48 (K48)-linked, or Lys63 (K63)-linked ubiquitinated FLNA. The following plasmids were used: HA Ub, HA-K48 Ub and HA-K63 Ub plasmids; a His-FLNA plasmid; and Flag-TRIM44 and Flag-vector plasmids. The cell extracts were immunoprecipitated by a His antibody. K48- and K63-polyubiquitinated FLNA were detected by Western blotting with the HA antibody. In Table S3, the antibodies applied in IP assay are presented.

### Animal experiments

The BALB/c nude mouse experiments were stringently approved by the Committee on the Ethics of Animal Experiments of Harbin Medical University. The operations were performed in accordance with the Guide for the Care Use of Laboratory Animals of the Harbin Medical University Institutional Animal Care and Use Committee.

In brief, a total of 5 × 10^6^ A549/DDP cells stably transfected with shTRIM44 and shNC were subcutaneously injected into each armpit of nude female mice. The mice were administered cisplatin at a dosage of 5 mg/kg intraperitoneally every 3 days after tumors were visible (100 mm^3^). Twenty-one days after the drug injection, the mice were sacrificed, and all tumors were excised, measured and weighed.

For further mechanistic studies *in vivo*, 30 mice were randomly divided into 6 groups and given different cell injections derived from A549 cells. The mice were grouped as follows: (I) Ctrl, (II) TRIM44, (III) TRIM44+Vector, (IV) TRIM44+shBRCA1, (V) TRIM44+Vector, and (VI) TRIM44+shFLNA. The subsequent operations were carried out in accordance with the above protocols.

### Immunohistochemistry (IHC)

IHC assays were carried out as reported previously 62. The antibodies used are presented in Table S3.

### Tissue specimens

All LUAD tissues (n=100) from 50 cisplatin-sensitive and 50 cisplatin-resistant patients in this study were collected at Harbin Medical University Cancer Hospital. The study was conducted after approval by the Ethical Review Committee of Harbin Medical University Cancer Hospital.

### Statistical analysis

Briefly, Student's t test was applied to assess the statistical significance of differences between two groups for normally distributed continuous data, and *P* < 0.05 indicated significance. All statistical analyses were conducted using GraphPad Prism 8.0.2 software.

## Supplementary Material

Supplementary figures.

## Abbreviations

LUAD: lung adenocarcinoma
NSCLC: non-small cell lung cancer
CDDP: cis-diamminedichloroplatinum II
TRIM44: Tripartite motif-containing 44
BRCA1: Breast cancer susceptibility gene 1
FLNA: filamin A
HR: homologous recombination
DDR: DNA damage response
DSBs: double-strand breaks
TCGA: The Cancer Genome Atlas
GO: gene ontology
DEGs: differentially expressed genes
IF: Immunofluorescence
IP: immunoprecipitation
CHX: cycloheximide
qRT-PCR: quantitative real-time PCR
CCK-8: Cell Counting Kit-8
IHC: immunohistochemistry
