# Supplementary material for: TRIM44 promotes BRCA1 functions in HR repair to induce Cisplatin Chemoresistance in Lung Adenocarcinoma by Deubiquitinating FLNA
Source: Int J Biol Sci. 2022 Apr 18;18(7):2962–79. doi: 10.7150/ijbs.71283 (PMC9066100; doi:10.7150/ijbs.71283)
Supplement: Supplementary file 1 — Supplementary figures. [file ijbsv18p2962s1.pdf]

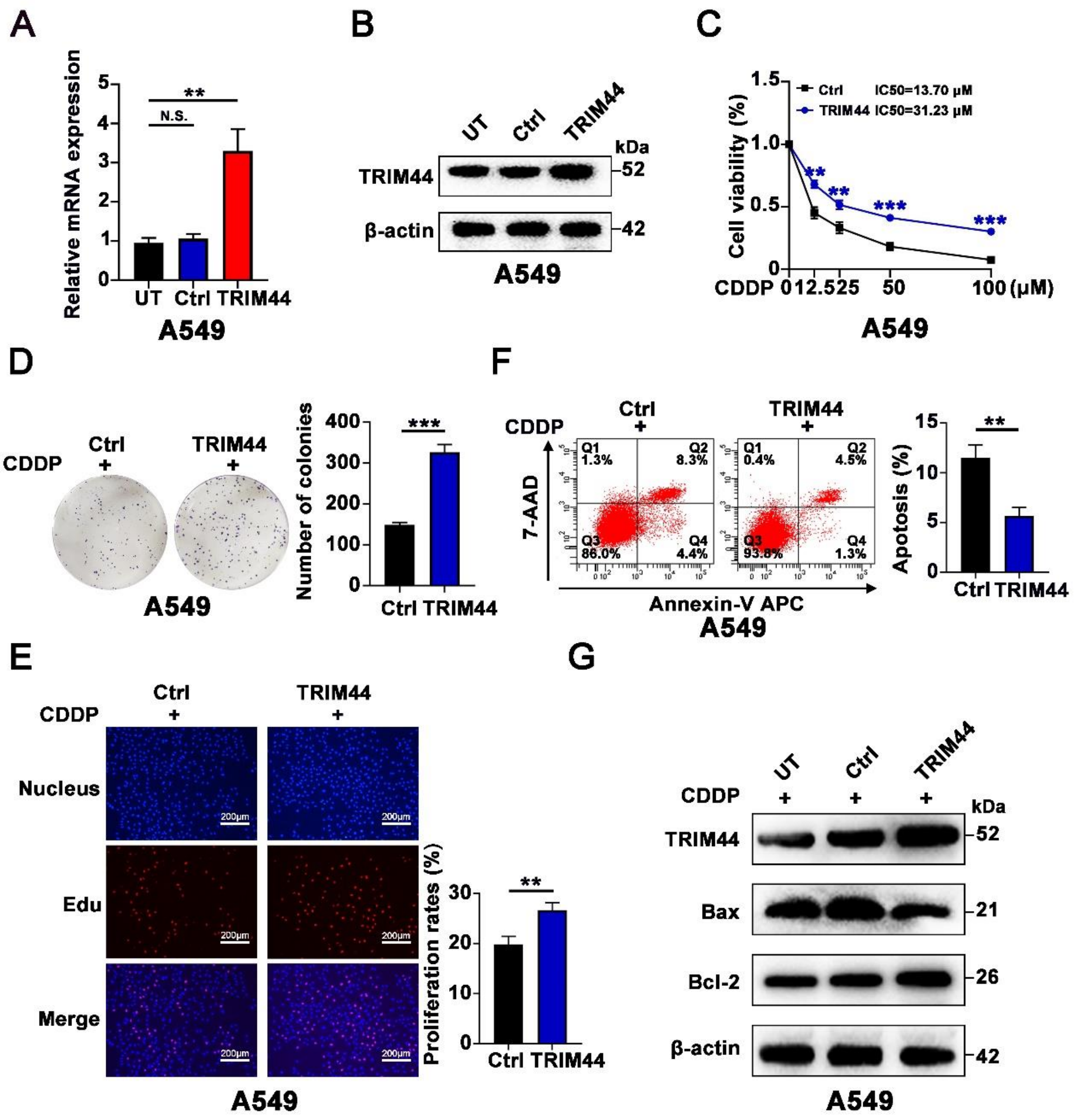

Figure S1. TRIM44 overexpression induces cisplatin resistance in LUAD cells. (A-B) TRIM44 expression was determined using qRT–PCR (A) and Western blotting (B) in TRIM44-overexpressing clones (TRIM44) and a control-expressing clone (Ctrl) derived from A549 cells. **(C)** The effect of TRIM44 overexpression on the viability of A549 cells in the presence of cisplatin. **(D-E)** Plate colony formation (D) and EdU (E) assays were performed to evaluate the proliferation capacity of Ctrl and TRIM44 cells derived from A549 cells exposed to cisplatin. **(F)** Evaluation of cisplatin-induced apoptosis in the designated cells using flow cytometry. **(G)** Expression of apoptosis-

related markers after TRIM44 overexpression in A549 cells. Data are shown as the mean ± SD. $P > 0.05$ was considered not significant (N.S.), $**P < 0.01$, and $***P < 0.001$.

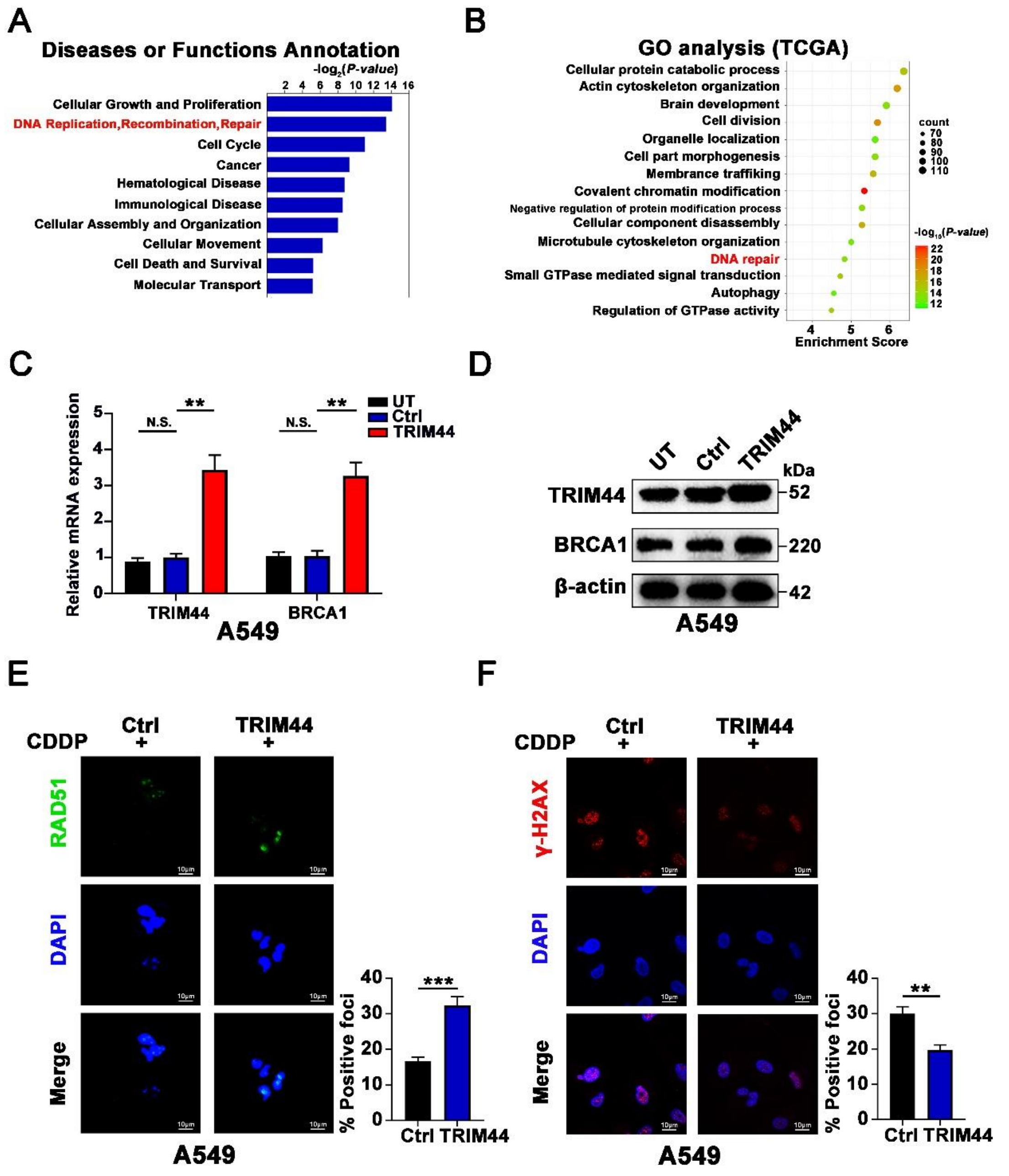

Figure S2. TRIM44 overexpression promotes BRCA1 expression and the effect of BRCA1 on HR. (A) The "*Disease or Functions Annotation*" of the IPA software was performed to summarize the enrichment of differential genes between NC or shTRIM44.

All functions were sorted by the $-Log_2$ (*P value*). **(B)** The putative biological role of TRIM44 was investigated using gene ontology (GO) enrichment analysis. **(C-D)** TRIM44 overexpression promoted BRCA1 mRNA (C) and protein (D) expression. **(E-F)** A549-TRIM44 cells exhibited more RAD51 foci (E) and fewer γ-H2AX foci (F) than that of A549-Ctrl cells when treated with cisplatin. (E) Representative immunofluorescence images showing RAD51 foci (left panel) and bar graphs showing the statistical analysis (right panel). (F) Representative immunofluorescence images showing γ-H2AX foci (left panel) and bar graphs showing the statistical analysis (right panel). Data are shown as the mean ± SD. $P > 0.05$ was considered not significant (N.S.), $^{**}P < 0.01$, and $^{***}P < 0.001$.

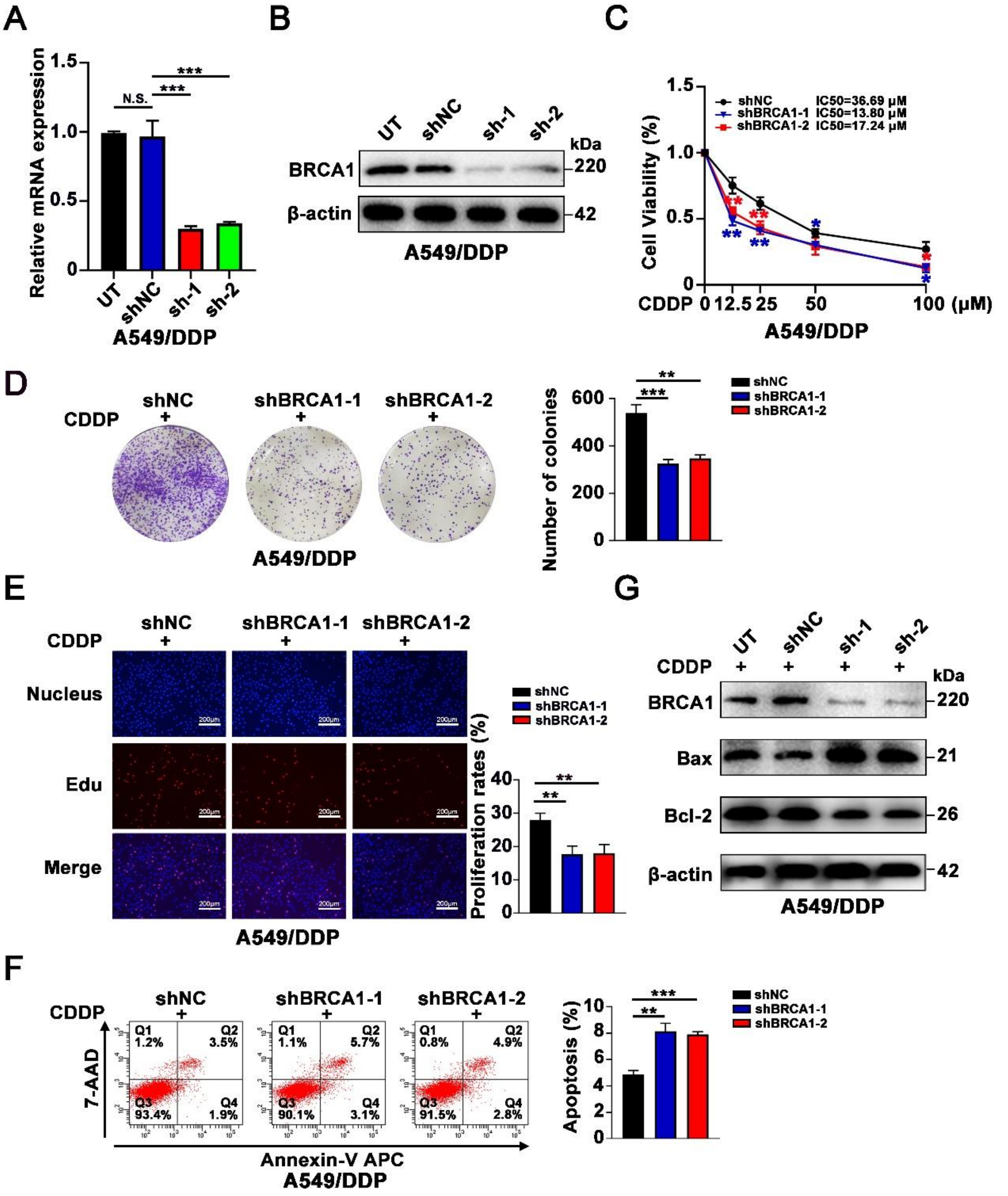

Figure S3. BRCA1 knockdown makes cisplatin-resistant LUAD cells susceptible to cisplatin. (A-B) BRCA1 expression in A549/DDP cells transfected with shNC, shBRCA1-1, or shBRCA1-2 was determined by qRT–PCR (A) and Western blotting (B). **(C)** CCK-8 analysis showed the viability of shNC, shBRCA1-1 and shBRCA1-2 derived by A549/DDP cells after being treated for 48 h with cisplatin. **(D)** The indicated cells were treated with cisplatin for 14 days at a dosage of 10 μM. Colonies were stained

with crystal violet (left panel). The bar graphs show the statistical analysis of the number of colonies (right panel). **(E)** EdU assay of A549/DDP cells transfected with shNC or BRCA1 shRNAs in the presence of cisplatin (10 μM). **(F-G)** Apoptosis analysis of the indicated A549/DDP-derived cells treated with cisplatin was performed by flow cytometric analysis (F) and Western blotting (G). Data are shown as the mean ± SD. $P > 0.05$ was considered not significant (N.S.), $*P < 0.05$, $**P < 0.01$ and $***P < 0.001$.

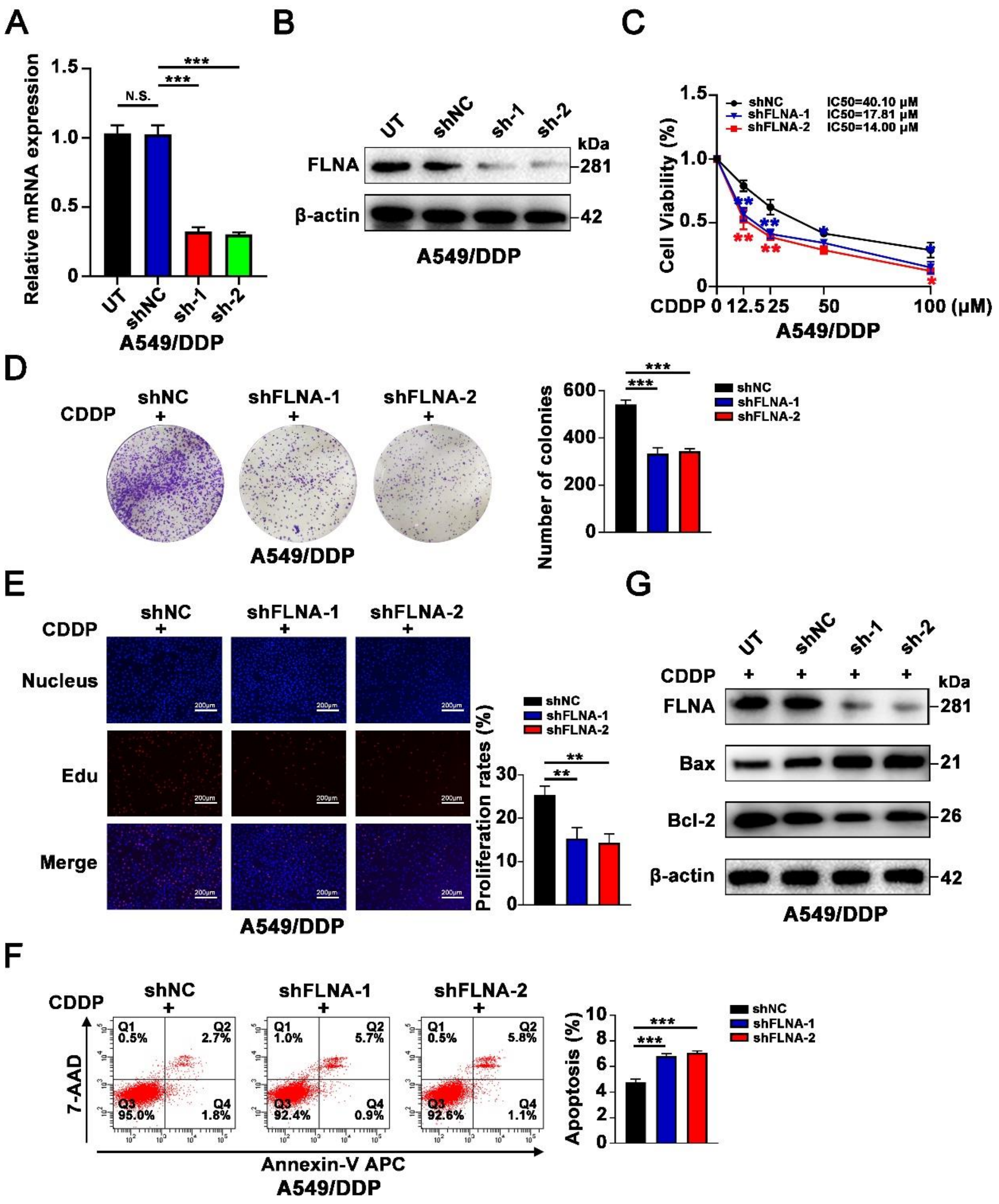

Figure S4. FLNA depletion makes cisplatin-resistant LUAD cells susceptible to cisplatin. (A-B) FLNA expression in A549/DDP cells transfected with shNC, shFLNA-1, or shFLNA-2 was determined by qRT–PCR (A) and Western blotting (B). **(C-E)** The influence of FLNA silencing on the viability (C), colony formation (D), and proliferation (E) of A549/DDP cells following treatment with cisplatin. **(F-G)** The effect of FLNA knockdown on cisplatin-induced apoptosis was assessed by flow

cytometry (F) and Western blotting for the apoptosis-related markers (G). Data are shown as the mean ± SD. $P > 0.05$ was considered not significant (N.S.), $*P < 0.05$, $**P < 0.01$ and $***P < 0.001$.

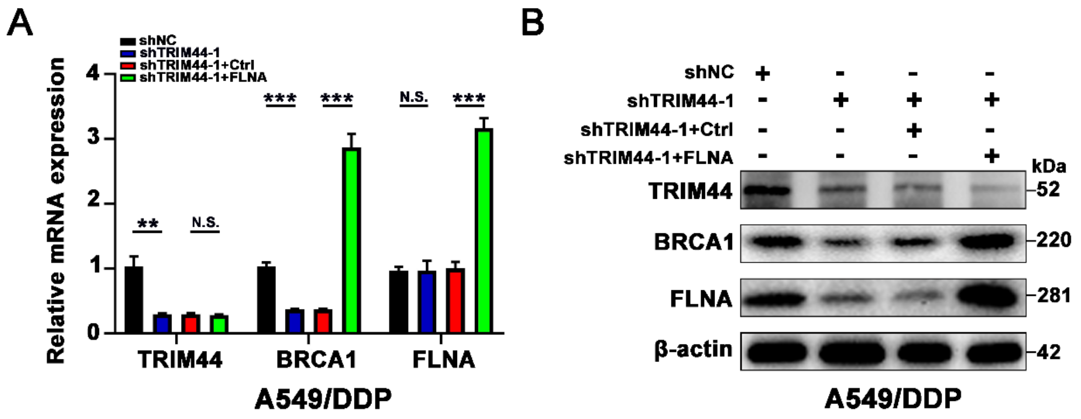

Figure S5. FLNA is required for TRIM44 knockdown inhibiting BRCA1 expression. A549 cells were transfected with lentivirus expressing either NC, shTRIM44-1, shTRIM44-1+Ctrl, or shTRIM44-1+FLNA. **(A-B)** The expression of BRCA1 was reduced following FLNA transfection into TRIM44-knockdown A549/DDP cells, according to qRT–PCR (A) and Western blotting (B) analysis. Data are shown as the mean ± SD. $P > 0.05$ was considered not significant (N.S.), $**P < 0.01$, and $***P < 0.001$.

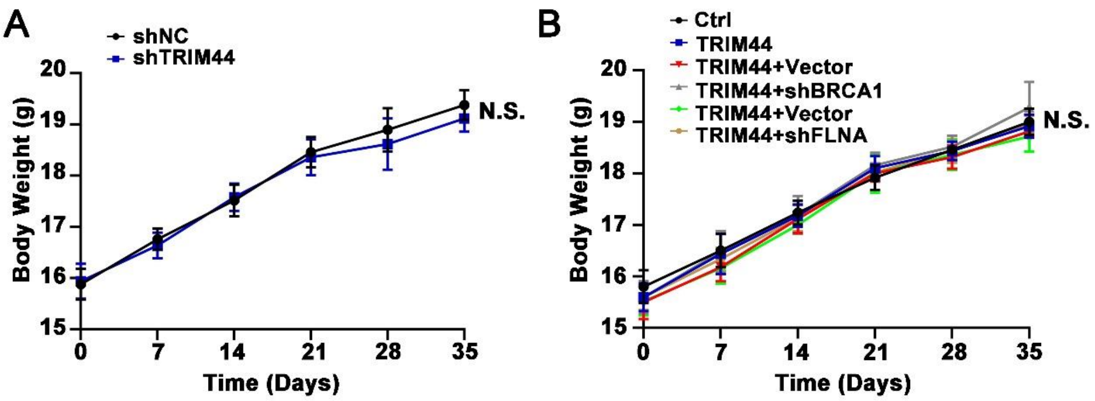

Figure S6. The average body weights of the mice. (A) The average body weights of

mice in the NC and shTRIM44 groups were statistically analyzed. **(B)** The average body weights of the mice in the different groups were statistically analyzed. Data are shown as the mean ± SD. $P > 0.05$ was considered not significant (N.S.).
